# Dietary fat increases solid tumor growth and metastasis of 4T1 murine mammary carcinoma cells and mortality in obesity-resistant BALB/c mice

**DOI:** 10.1186/bcr2927

**Published:** 2011-08-11

**Authors:** Eun Ji Kim, Mi-Ran Choi, Heesook Park, Minhee Kim, Ji Eun Hong, Jae-Yong Lee, Hyang Sook Chun, Ki Won Lee, Jung Han Yoon Park

**Affiliations:** 1 Department of Food Science and Nutrition, College of Natural Sciences, Hallym University, 39 Hallymdaehak-gil, Chuncheon, 200-702, Korea; 2 Center for Efficacy Assessment and Development of Functional Foods and Drugs, Hallym University, 39 Hallymdaehak-gil, Chuncheon, 200-702, Korea; 3 Department of Biochemistry, College of Medicine, Hallym University, 39 Hallymdaehak-gil, Chuncheon, 200-702, Korea; 4 Korea Food Research Institute, 516 Baekhyun-dong, Bundang-gu, Sungnam, 463-746, Korea; 5 Department of Agricultural Biotechnology and Center for Agricultural Biomaterials, Seoul National University, 1 Gwanak-ro, Gwanak-gu, Seoul, 151-921, Korea

## Abstract

**Introduction:**

High-fat diets (HFDs) are known to cause obesity and are associated with breast cancer progression and metastasis. Because obesity is associated with breast cancer progression, it is important to determine whether dietary fat *per se *stimulates breast cancer progression in the absence of obesity. This study investigated whether an HFD increases breast cancer growth and metastasis, as well as mortality, in obesity-resistant BALB/c mice.

**Methods:**

The 4-week-old, female BALB/c mice were fed HFD (60% kcal fat) or control diet (CD, 10% kcal fat) for 16 weeks. Subsequently, 4T1 mammary carcinoma cells were injected into the inguinal mammary fat pads of mice fed continuously on their respective diets. Cell-cycle progression, angiogenesis, and immune cells in tumor tissues, proteases and adhesion molecules in the lungs, and serum cytokine levels were analyzed with immunohistochemistry, Western blotting, and enzyme-linked immunosorbent assay (ELISA). *In vitro *studies were also conducted to evaluate the effects of cytokines on 4T1 cell viability, migration, and adhesion.

**Results:**

Spleen and gonadal fat-pad weights, tumor weight, the number and volume of tumor nodules in the lung and liver, and tumor-associated mortality were increased in the HFD group, with only slight increases in energy intake and body weight. HF feeding increased macrophage infiltration into adipose tissues, the number of lipid vacuoles and the expression of cyclin-dependent kinase (CDK)2, cyclin D1, cyclin A, Ki67, CD31, CD45, and CD68 in the tumor tissues, and elevated serum levels of complement fragment 5a (C5a), interleukin (IL)-16, macrophage colony-stimulating factor (M-CSF), soluble intercellular adhesion molecule (sICAM)-1, tissue inhibitors of metalloproteinase (TIMP)-1, leptin, and triggering receptor expressed on myeloid cells (TREM)-1. Protein levels of the urokinase-type plasminogen activator, ICAM-1, and vascular cell adhesion molecule-1 were increased, but plasminogen activator inhibitor-1 levels were decreased in the lungs of the HFD group. *In vitro *assays using 4T1 cells showed that sICAM-1 increased viability; TREM-1, TIMP-1, M-CSF, and sICAM-1 increased migration; and C5a, sICAM-1, IL-16, M-CSF, TIMP-1, and TREM-1 increased adhesion.

**Conclusions:**

Dietary fat increases mammary tumor growth and metastasis, thereby increasing mortality in obesity-resistant mice.

## Introduction

Breast cancer is a leading cause of cancer-associated mortality in women in the United States [1], and the incidence is increasing in the developing world. The majority of breast cancer-related death results from uncontrolled metastatic disease. Although in 10% to 15% of cases, breast cancer spreads to other parts of the body within 3 years of initial diagnosis, metastasis tends to recur later, 10 years or more after the detection of the primary tumor [2]. However, current therapies including surgery, hormone therapy, chemotherapy, radiation therapy, and selective combinations thereof are not completely effective in the treatment of metastatic breast cancer [3]. Thus, it is important to find safe and effective lifestyle modifications, including dietary habits, for decreasing breast cancer development and metastasis.

Epidemiologic studies indicate that consuming a high-fat (regardless of fat type) diet may lead to an increased risk of invasive breast cancer in postmenopausal women [4]. High-fat diets are known to induce obesity in humans and rodents [5,6], and obese women have an increased risk of developing postmenopausal breast cancer [7,8]. Additionally, a high body mass index (BMI) is associated with poor prognosis in breast cancer patients [9,10]. Thus, it is clearly important to understand the molecular basis for the association of high-fat diet and/or obesity with breast cancer development and mortality.

In 1991 Rose *et al*. [11] reported that a diet containing 23% as opposed to 5% (wt/wt) corn oil (rich in the omega-6 fatty acid linoleic acid) increased the tumor growth rate and lung-metastasis incidence of MDA-MB-435 human breast cancer cells injected into the mammary fat pads of athymic nude mice. To evaluate the effects of a high-fat diet on cancer development and progression and the underlying mechanisms thereof, we used the 4T1 orthotopic model, in which 4T1 mammary carcinoma cells are injected into the mammary fat pads of immune-competent BALB/c mice. The 4T1 cells were derived from the mammary tumors of BALB/c mice lacking protein expression of the estrogen receptor α [12,13]. When injected into the mammary fat pads of syngeneic BALB/c mice, 4T1 cells grow into solid tumors that metastasize to the lung, liver, lymph nodes, and brain, while the primary tumor grows *in situ *[14,15]. The 4T1 orthotopic model closely mimics the progressive forms of estrogen-insensitive human metastatic breast cancer [16]. Additionally, BALB/c mice are obesity resistant, and high-fat diet (HFD) consumption has little effect on body weight [17].

In this study we demonstrated that the prolonged consumption of an HFD without any reduction in the intake of protein, minerals, vitamins, and fiber has little effect on energy intake and body weight but does increase breast cancer growth and metastasis, as well as mortality in BALB/c mice.

## Materials and methods

### Materials

Reagents were purchased from the following suppliers: 3-[4,5-dimethylthiazol-2-yl]-2,5-diphenyltetrazolium bromide (MTT), Bouin solution, and anti-β-actin antibody from Sigma (St. Louis, MO, USA); antibodies against CD45, matrix metalloproteinase (MMP)-9, tissue inhibitor of matrix metalloproteinase (TIMP)-2, intercellular adhesion molecule (ICAM)-1, and vascular cell adhesion molecule (VCAM)-1, and enzyme-linked immunosorbent assay (ELISA) kits for soluble ICAM-1, macrophage colony stimulating factor (M-CSF), TIMP-1, leptin, and triggering receptor expressed on myeloid cells (TREM)-1 from R and D Systems (Minneapolis, MN, USA); ELISA kits for complement fragment 5a (C5a) and interleukin (IL)-16 from USCN Life Science and Technology Company (Missouri City, TX, USA); antibodies against urokinase-type plasminogen activator (uPA) from Calbiochem (La Jolla, CA, USA); antibodies against E2F1, proliferating cell nuclear antigen (PCNA), p27, cyclin-dependent kinase (CDK)2, CDK4, cyclin A, cyclin D1, CD31, and vascular endothelial growth factor (VEGF), plasminogen activator inhibitor (PAI)-1 from Santa Cruz Biotechnology (Santa Cruz, CA, USA); antibodies against Ki67 and F4/80 from Abcam (Cambridge, MA, USA); horseradish peroxidase-conjugated anti-rabbit, anti-mouse, and anti-goat IgG from Amersham Biosciences (Arlington Heights, IL, USA); an antibody against CD68 from ABBIOTEC (San Diego, CA, USA); and Immobilon Western Chemiluminescent HRP Substrate and adhesion assay kit from Millipore Corporation (Billerica, MA, USA). If not otherwise noted, all other materials were purchased from Sigma-Aldrich Co.

### 4T1 cell culture

4T1 murine mammary carcinoma cells were acquired from the American Type Culture Collection (Manassas, MA, USA) and maintained in Dulbecco's Modified Eagle's Medium (DMEM) containing 100 ml/L of fetal bovine serum (FBS) with 100,000 U/L of penicillin and 100 mg/L of streptomycin in a humidified atmosphere of 5% CO_2 _in air.

### Animals

Three-week-old female BALB/c mice were purchased from Orient Bio Inc. (Seongnam, Korea) and housed at the animal research facility of Hallym University. Mice were acclimatized to the laboratory conditions and provided free access to a standard nonpurified rodent diet (Superfeed Co., Wonju, Korea) and water. The mice were acclimated for 1 week before use and maintained throughout the study in a controlled environment: 24 ± 2°C, 50 ± 10% relative humidity, and a 12-hour light/dark cycle. To determine the survival rate of animals, mice were killed when they reached moribund conditions, as described by Toth *et al*. [18]. All experiments were conducted in accordance with the protocols approved by the Animal Care and Use Committee of the Hallym University, Korea (Ethical approval number: Hallym 2009-122).

### Diets

The mice were randomly divided into two groups; the control and high-fat groups. The purified diets used in this study were purchased from Research Diets, Inc. (New Brunswick, NJ, USA). The control diet (CD; No. D12450B) contained 10% of kcal from fat, and the HFDs (No. D12451 and D12452) contained 45% and 60% of kcal from fat. The lard content was 4.4, 39.4, and 54.4 kcal% in the 10, 45, and 60 kcal% diets, respectively. The three diets contained identical quantities of protein, cellulose, soybean oil, vitamins, and minerals per kilocalorie (Additional file 1). Fresh diet was freely provided each day, and daily feed intake was monitored throughout the study.

### *In vivo *mammary cancer orthograft model

Twelve or 16 weeks after initiating feeding, 4T1 cells (5 × 10^4 ^cells suspended in 0.1 ml Matrigel(BD Biosciences, San Jose, CA, USA) were injected into the inguinal mammary fat pads of the mice. The mice continued on their respective diets. In experiment I, the mice were maintained for 42 days after the 4T1 cell injection to monitor mammary cancer-related death, and the Kaplan-Meier curve was plotted for two dietary groups associated with mouse survival. In experiment II, 25 days after the 4T1 cell injections, the mice were killed with carbon dioxide asphyxiation, and the tumors, lungs, livers, kidneys, gonadal fat pads, and spleens were excised from the mice and weighed. The sera were prepared for ELISA.

Tumor volumes were measured with a set of calipers and calculated by using the following formula: 0.52 × long diameter × short diameter^2 ^[19]. The tumors and gonadal fat pads were formalin fixed and paraffin embedded for immunohistochemistry (IHC) or homogenized to prepare the tissue lysates [20] for Western blot analysis. The lungs and livers were fixed in Bouin solution or homogenized to prepare the tissue lysates [20]. Metastatic nodules in the lungs and livers were counted, and the total tumor volumes were estimated as described previously [21,22].

### Immunohistochemistry

Paraffin-embedded tumor tissues were sectioned, deparaffinized, rehydrated, incubated in 3% H_2_O_2_, and blocked with 5% BSA, as previously described [23]. IHC was conducted with the indicated antibodies, biotinylated rabbit anti-mouse IgG, streptavidin-horseradish peroxidase, 1,3 -diaminobenzidine (DAB) tetrahydrochloride, and hematoxylin, as described previously [23]. Randomly chosen fields were photographed at ×200 magnification, and immuno-positive cells and staining intensities were quantified with a Carl Zeiss AxioImager microscope and Image M1 Software (Carl Zeiss, Jena, Germany).

### Western blot analysis

Western blot analyses were conducted as described previously [24]. Signals were detected through enhanced chemiluminescence by using Immobilon Western Chemiluminescent HRP Substrate (Millipore Corporation). The relative abundance of each band was quantified by using the Bio-profile Bio-1D application (Vilber-Lourmat, Marine la Vallee, France), and the expression levels were normalized to β-actin.

### Mouse cytokine antibody array

Pooled sera from each group were applied to a proteome profiler antibody array kit (R&D Systems) to evaluate cytokine expression, in accordance with the manufacturer's instructions. The relative abundance of each protein was quantified by using the Bio-profile Bio-1D application (Vilber-Lourmat), and the expression levels were normalized to the control protein.

### Enzyme-linked immunosorbent assay

The levels of C5a, sICAM-1, IL-16, M-CSF, TIMP-1, leptin, and TREM-1 in sera were estimated by using the relevant ELISA kits according to the manufacturers' instructions.

### Cell-viability assay

4T1 cells were plated in 24-well plates at 5 × 10^4 ^cells/well in DMEM supplemented with 100 ml/L FBS. One day later, the monolayers were serum deprived with DMEM supplemented with 10 ml/L charcoal-stripped FBS (serum-deprivation medium) for 24 hours, and the cells were incubated in serum-deprivation medium in the absence or presence of various cytokines. Viable cell numbers were estimated with an MTT assay, as described previously [25].

### *In vitro *migration assay

The cell-migration assay was conducted as described previously [26]. Serum-deprived cells were plated onto the filter in 6.5-mm transwell inserts in 24-well plates at 5 × 10^4 ^cells/filter and treated for 16 hours with various cytokines in serum-deprivation medium. Migrated cells were stained with hematoxylin and eosin (H&E), and then counted under a light microscope in eight randomized fields.

### Adhesion assay

4T1 cells were plated in human collagen type I-coated CytoMatrix Cell Adhesion Strips at 1 × 10^5 ^cells. Cells were incubated in DMEM containing 10 ml/L of charcoal-stripped FBS with various cytokines at 37°C for 45 minutes. Cells were stained with crystal violet, and the cell-bound stains were quantified by measuring the absorbance at 570 nm, as described previously [26].

### Statistical analysis

The data were expressed as the mean ± SEM. The significance of the difference between groups was evaluated with the Student *t *test, by using SAS for Windows version 9.1 (SAS Institute). Differences were considered significant at *P *< 0.05. The Kaplan-Meier curves were analyzed with a Log-Rank test to assess the significance of the differences in the survival rates of the mice.

## Results

### Prolonged high-fat diet consumption increases mammary cancer-related mortality in BALB/c mice injected with 4T1 cells

**Figure 1:**
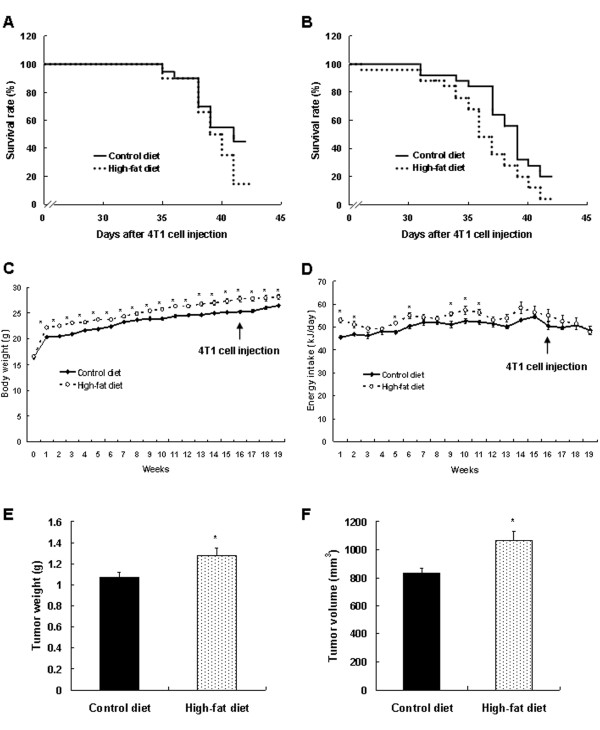
**A high-fat diet reduces the survival rate and increases solid tumor growth in BALB/c mice injected with 4T1 cells**. **(a) **Four-week-old, female BALB/c mice were fed on a high-fat diet in which 45% of the kilocalories were provided as fats or a control diet in which 10% of kilocalories were provided as fats for 12 weeks. Twelve weeks after initiating feeding, 4T1 cells (5 × 10^4 ^cells suspended in 0.1 ml gelatinous protein (Matrigel) were injected into the inguinal mammary fat pads of the mice. The mice were fed continuously on the same diets. Survival rates were monitored after the 4T1 cell injection (0 day). **(b) **Four-week-old, female BALB/c mice were fed on a high-fat diet in which 60% of the kilocalories were provided as fats or the control diet (10% kcal fat) for 16 weeks. Sixteen weeks after the initiation of feeding, 4T1 cells were injected, and mice were continuously fed on the same diet. **(c-f) **Mice were fed on the diets and injected with 4T1 cells, as described in **b**, except that they were killed 25 days after the 4T1 cells injection. **(c) **Body weights of mice were measured every week. Each point of body weight represents the mean ± SEM (*n *= 30). **(d) **Energy intake was calculated on the basis of 16.12 kJ/g in the control diet and 21.93 kJ/g in the high-fat diet. Each point of energy intake represents the mean ± SEM (*n *= 6). *Significantly different from the control group, *P *< 0.05. **(e) **The tumors were excised from mice and weighed. **(f) **The tumor volume was measured by using calipers and calculated with the formula: 0.52 × long diameter × short diameter^2^. Each bar represents the mean ± SEM (*n *= 30). *Significantly different from the control group, *P *< 0.05.

The first study determined mortality rates in 4T1 cell-injected mice fed HFD (45%). Relative to the mice fed on the CD (10% kcal as fats), the survival rate was lower in the mice fed HFD (*P *< 0.001; Figure 1a). In the subsequent study, the mice were fed on a 60% kcal-diet or the CD for 16 weeks and then injected with 4T1 cells. Again, survival rates were reduced in the mice fed on the HFD (60% kcal) as compared with controls (*P *< 0.001, Figure 1b).

### Long-term consumption of a 60% kcal-fat diet increases solid tumor growth of 4T1 cells in BALB/c mice

Body weights were slightly higher in the mice fed on the 60% kcal-fat diet than in controls from 1 week onward after initiating experimental diets (Figure 1c). Final body weights were 24.7 ± 0.3 g and 26.2 ± 0.5 g in the control and high-fat groups. Energy intakes were only slightly higher in the high-fat group (Figure 1d). Spleen and gonadal fat-pad weights were significantly higher in the HFD-fed mice. However, the weights of the liver, lung, and kidney were unaffected by prolonged feeding on the HFD (Table 1). The subcutaneous fat, mesenteric fat, and retroperitoneal fat were almost undetectable to the naked eye in the BALB/c mice fed on either the CD or HFD. The wet weight of the primary solid tumors was increased by 22.4% (*P *< 0.0047), and tumor volume, increased by 28.0% (*P *< 0.0022) in HFD-fed mice compared with controls (Figure 1e, f).

**Table 1:** Effect of chronic consumption of a high-fat diet on organ weights in BALB/c mice injected with 4T1 cells

	Control diet	High-fat diet
Liver (g)	1.34 ± 0.02	1.35 ± 0.03
Lung (g)	0.28 ± 0.01	0.30 ± 0.01
Kidney, right (g)	0.16 ± 0.01	0.16 ± 0.01
Kidney, left (g)	0.16 ± 0.01	0.16 ± 0.01
Spleen (g)	0.81 ± 0.04	1.02 ± 0.04^a^
Gonadal fat pad (g)	0.19 ± 0.02	0.25 ± 0.04^a^

Mice were fed on the high-fat (60% kcal) or control diet (10% kcal) for 16 weeks and injected with 4T1 cells, as described in Materials and Methods. Twenty-five days after the injection of 4T1 cells, the mice were killed, and the lungs, livers, kidneys, spleens, and gonadal fat pads were excised from the mice and weighed. Values represent the means ± SEM (*n *= 30). ^a^Significantly different from the control group, *P *< 0.05.

### Long-term consumption of a 60% kcal-fat diet increases cancer cell proliferation, angiogenesis, and infiltration of immune cells in 4T1 tumors in BALB/c mice

**Figure 2:**
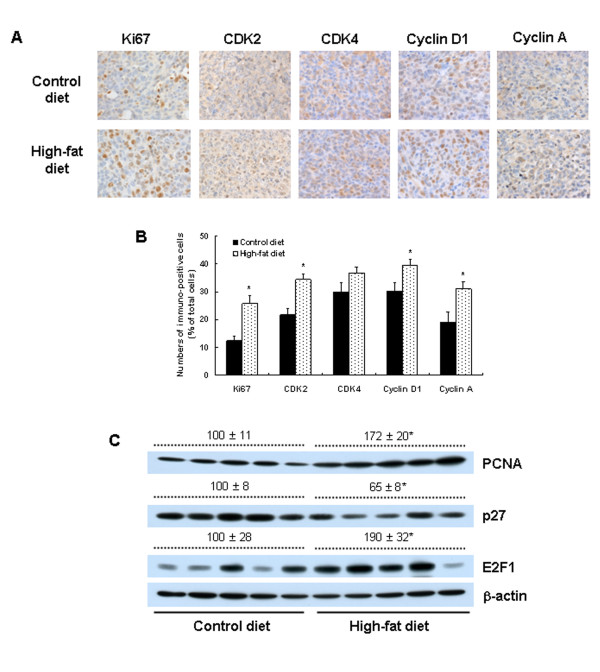
**Dietary fat stimulates cell-cycle progression in 4T1 tumors in BALB/c mice**. Mice were fed, injected with 4T1 cells, and then killed as described in Figure 1c. **(a) **Tumor sections were stained with antibody raised against Ki67, cyclin-dependent kinase (CDK)2, CDK4, cyclin D1, or cyclin A, and then with 1,3 -diaminobenzidine (DAB) and counterstained with hematoxylin (*n *= 15). Representative images of the immunohistochemical analysis are shown. **(b) **The Ki67-, CDK2-, CDK4-, cyclin D1-, and cyclin A-positive cells were counted. Each bar represents the means ± SEM (*n *= 15). **(c) **Tumor lysates were analyzed with Western blotting with the indicated antibodies. Photographs of chemiluminescent detection of the blots are shown. The relative abundance of each band to its own β-actin was quantified, and the control levels were at 100%. The adjusted mean ± SEM is shown above each blot. *Significantly different from the control group, *P *< 0.05. PCNA, proliferating cell nuclear antigen.

IHC staining revealed that the HFD-fed mice had significantly increased expression of Ki67 (*P *< 0.0084), CDK2 (*P *< 0.0152), cyclin D1 (*P *< 0.0381), and cyclin A (*P *< 0.0284) relative to controls (Figure 2a, b). Western blotting showed that the expression levels of PCNA and E2F1 were increased by 72.0% (*P *< 0.017) and 90.0% (*P *< 0.05), respectively, in the tumor tissues of HFD-fed mice relative to controls. In contrast, p27 (Kip1) expression was reduced significantly in the HFD-fed mice (Figure 2c).

**Figure 3:**
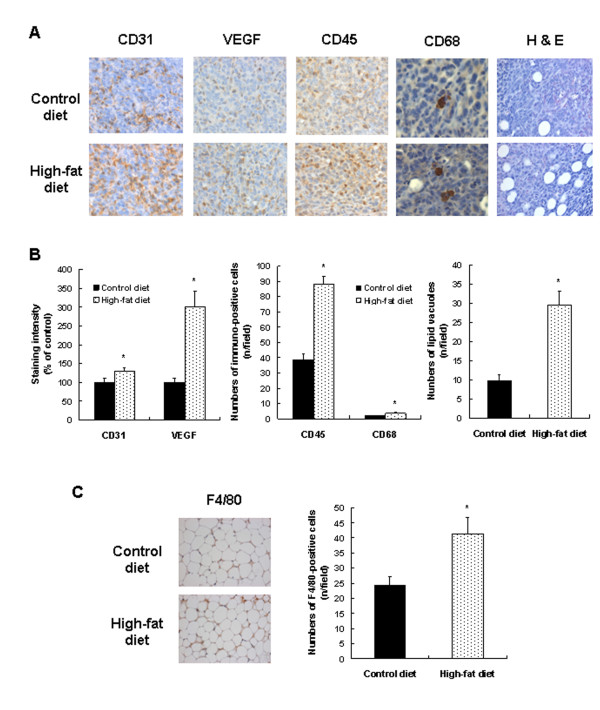
**Dietary fat stimulates angiogenesis and immune cell infiltration in 4T1 tumors and macrophage infiltration in the gonadal fat pad in BALB/c mice**. Mice were fed, injected with 4T1 cells, and then killed as described in Figure 1c. **(a) **Tumor sections were stained with antibody raised against CD31, vascular endothelial growth factor (VEGF), CD45, or CD68, and then with 1,3 -diaminobenzidine (DAB), and counterstained with hematoxylin, except that the last one was stained with hematoxylin and eosin (H&E) to visualize lipid vacuoles. Representative images of the immuno-histochemical analysis and H&E staining are shown, *n *= 15. **(b) **The staining intensity of CD31 and VEGF was quantified. CD45+ and CD68+ cells and the number of lipid vacuoles were counted. Each bar represents the mean ± SEM (*n *= 15). **(c) **Gonadal fat-pad sections were stained with an F4/80 antibody, and F4/80+ cells were counted, *n *= 15. Each bar represents the mean ± SEM (*n *= 15).*Significantly different from the control group, *P *< 0.05.

IHC staining showed that the expressions of CD31, VEGF, CD68, and CD45 were markedly increased in the tumors of the HFD-fed mice compared with controls (Figure 3a,b). H&E staining showed that the number of lipid vacuoles increased in the tumor tissues of the HFD-fed mice (Figure 3a, b). The number of F4/80+ cells was significantly increased in the gonadal fat pads of mice fed on the HFD (Figure 3c).

### Prolonged consumption of a 60% kcal-fat diet increases lung and liver metastasis of 4T1 cells in BALB/c mice

**Figure 4:**
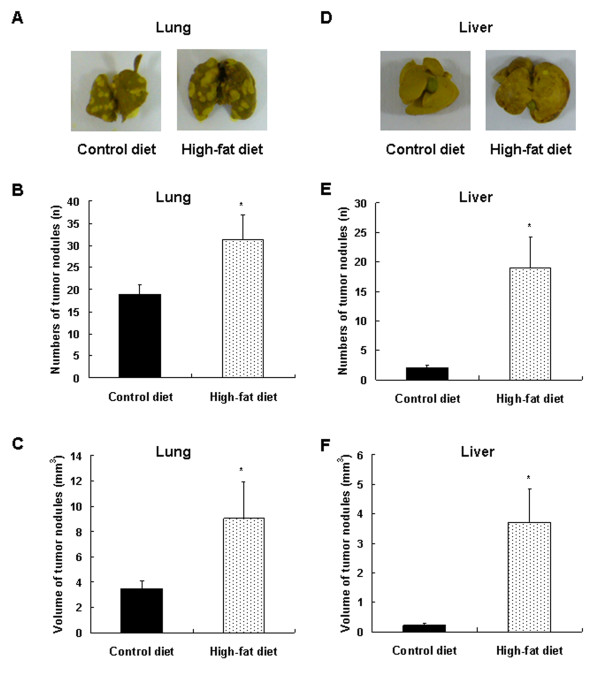
**Dietary fat stimulates the lung and liver metastasis of 4T1 cells in BALB/c mice**. Mice were fed, injected with 4T1 cells, and then killed as described in Figure 1c. The lungs and livers were excised from mice and fixed in Bouin solution. The numbers of tumor nodules in the lung and liver was counted, and the total tumor volumes were estimated. **(a, d) **Photographs of the lungs and livers, which were representative of 15 animals, are shown. Whitish tumor lesions can be observed on the surfaces of the lungs and livers. **(b, e) **The numbers of tumor nodules in the lung and liver. **(c, f) **The volume of tumor nodules in the lung and liver. Each bar represents the mean ± SEM (*n *= 15). *Significantly different from the control group, *P *< 0.05.

Tumor nodules grew in all mice in both groups, but in HFD-fed mice, the number and volume of tumor nodules on the lung increased by 65.2% (*P *< 0.0298) and 159.9% (*P *< 0.05), respectively, as compared with the mice fed on the CD (Figure 4a-c). In the liver, the incidence of tumor nodules was 35.7% and 66.7% in the CD group and HFD groups, respectively; and the number of nodules was 844% (*P *< 0.0159) greater in the HFD-fed mice. Additionally, long-term consumption of the HFD resulted in a 1,568% (*P *< 0.0188) increase in the volume of tumor nodules in the liver as compared with controls (Figure 4d-f).

### A 60% kcal-fat diet alters the expression of proteins involved in metastasis in the lungs of BALB/c mice injected with 4T1 cells

**Figure 5:**
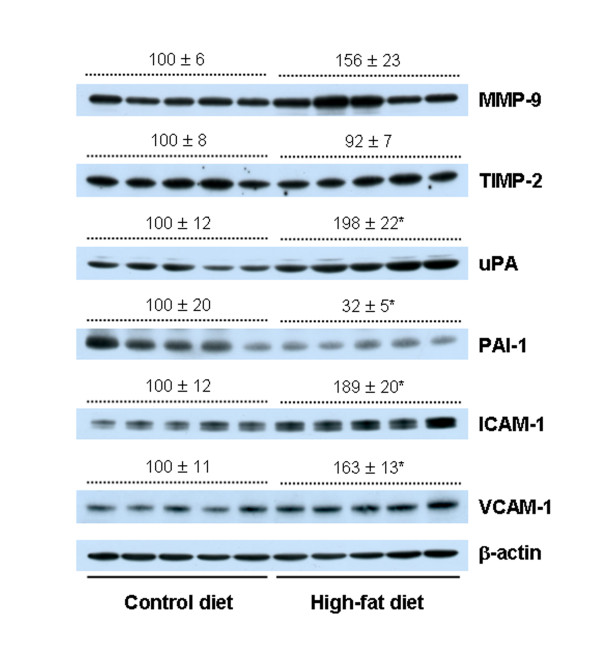
**Dietary fat alters the protein levels of urokinase-type plasminogen activator (uPA), plasminogen activator inhibitor (PAI)-1, intercellular adhesion molecule (ICAM)-1, and vascular cell adhesion molecule (VCAM)-1 in the lungs of BALB/c mice injected with 4T1 cells**. Mice were fed, injected with 4T1 cells, and then killed as described in Figure 1c. Lung lysates were analyzed with Western blotting with the indicated antibodies. Photographs of chemiluminescent detection of the blots are shown. The relative abundance of each band to its own β-actin was quantified, and the control levels were set at 100%. The adjusted mean ± SEM is shown above each blot. *Significantly different from the control group, *P *< 0.05. MMP, matrix metalloproteinase; TIMP, tissue inhibitor of matrix metalloproteinase.

Western blot analysis of lung tissue lysates revealed that HFD increased the protein levels of uPA, ICAM-1, and VCAM-1 in the lungs by 98.0% (*P *< 0.0089), 89.0% (*P *< 0.008), and 63.0% (*P *< 0.0069), respectively, as compared with control mice, whereas a significant (*P *< 0.05) reduction of PAI-1 expression was noted in the mice fed HFD (Figure 5).

### A 60% kcal-fat diet increases serum levels of C5a, sICAM-1, IL-16, M-CSF, TIMP-1, leptin, and TREM-1 in BALB/c mice injected with 4T1 cells

Because a variety of cytokines are known to affect the processes of breast cancer growth and metastasis (reviewed in [27]), the serum cytokines levels were estimated by using a mouse cytokine antibody array kit. Among the 40 cytokines measurable with the mouse cytokine array kit, seven cytokines (C5a, sICAM-1, IL-16, G-CSF, M-CSF, TIMP-1, and TREM-1) were detected in the sera of mice. The levels of C5a, sICAM-1, IL-16, M-CSF, TIMP-1, and TREM-1 were increased in the sera of the HFD-fed mice relative to those of the controls, whereas the level of granulocyte colony-stimulating factor (G-CSF) was not altered (data not shown). We subsequently confirmed the results with ELISA and noted changes similar to those observed in the antibody array. HFD increased serum levels of C5a, sICAM-1, IL-16, M-CSF, TIMP-1, and TREM-1 by 31.8% (*P *< 0.05), 31.8% (*P *< 0.0002), 26.0% (*P *< 0.002), 61.0% (*P *< 0.0001), 40.9% (*P *< 0.0032), and 41.3% (*P *< 0.002), respectively, relative to control mice. HFD also increased the serum levels of leptin by 36.0% (*P *< 0.0013) relative to controls (Table 2).

**Table 2:** Effect of prolonged consumption of a high-fat diet on the levels of various cytokines in the sera of BALB/c mice injected with 4T1 cells

	Control diet	High-fat diet
C5a (ng/ml)	22.0 ± 2.7	29.0 ± 3.1^a^
sICAM-1 (ng/ml)	494.2 ± 22.6	651.5 ± 29.4^a^
IL-16 (ng/ml)	150.8 ± 8.0	190.0 ± 8.4^a^
M-CSF (pg/ml)	714.1 ± 29.3	1,149.5 ± 35.6^a^
TIMP-1 (ng/ml)	4.4 ± 0.4	6.2 ± 0.4^a^
TREM-1 (pg/ml)	684.7 ± 17.8	967.8 ± 21.6^a^
Leptin (ng/ml)	138.0 ± 0.04	187.8 ± 11.3^a^

Mice were fed on the high-fat (60% kcal) or control diet (10% kcal) for 16 weeks and injected with 4T1 cells, as described in Materials and Methods. At 25 days after the 4T1 cells injection, blood samples were collected from the mice, and the sera were prepared. The serum levels of complement fragment 5a (C5a), soluble intercellular adhesion molecule (sICAM)-1, interleukin (IL)-16, macrophage colony-stimulating factor (M-CSF), tissue inhibitor of matrix metalloproteinase (TIMP)-1, triggering receptor expressed on myeloid cells (TREM)-1, and leptin were measured with the appropriate ELISA kits. Values represent the mean ± SEM (*n *= 30). ^a^Significantly different from the control group, *P *< 0.05.

### Various cytokines elevated by HFD increased the viability, adhesion, and migration of 4T1 cells *in vitro*

**Figure 6:**
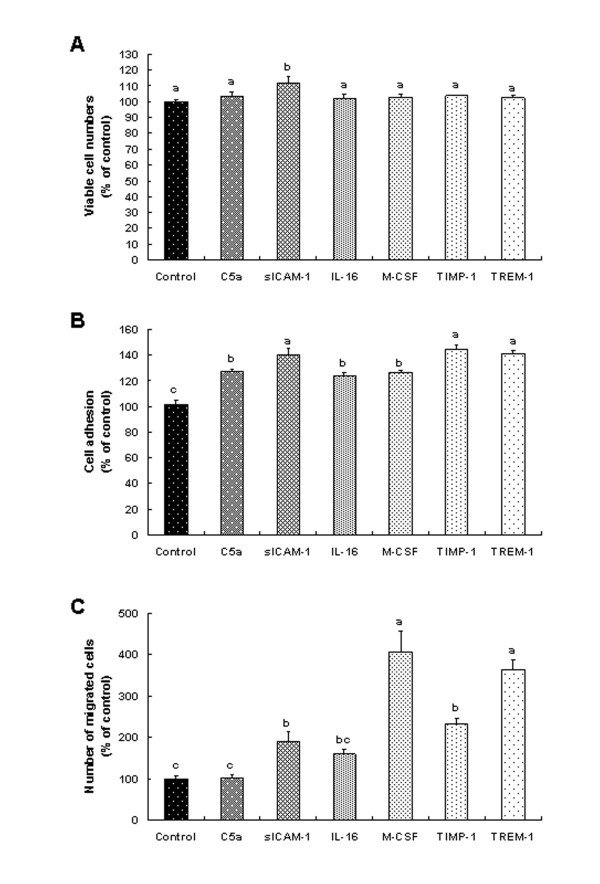
**Effect of various cytokines on proliferation (a), adhesion (b), and migration (c) of 4T1 cells *in vitro***. **(a) **4T1 cells were plated in 24-well plates at 5 × 10^4 ^cells/well in DMEM supplemented with 100 ml/L FBS. One day later, the monolayers were serum deprived with DMEM supplemented with 10 ml/L charcoal-stripped FBS (serum-deprivation medium) for 24 h. After serum deprivation, the cells were incubated in serum-deprivation medium in the absence or presence of 29 ng/ml complement fragment 5a (C5a), 652 ng/ml soluble intercellular adhesion molecule (sICAM)-1, 190 ng/ml interleukin (IL)-16, 1,150 pg/ml macrophage colony-stimulating factor (M-CSF), 6.2 ng/ml tissue inhibitor of matrix metalloproteinase (TIMP)-1, and 970 pg/ml triggering receptor expressed on myeloid cells (TREM)-1 for 24 hours. Viable cell numbers were estimated. Each bar represents the mean ± SEM (*n *= 3). Means without a common letter differ, *P *< 0.05. **(b) **Cells were plated in human collagen type I-coated CytoMatrix Cell Adhesion Strips and incubated with or without various cytokines (the identical concentrations of cytokines used in Figure 6a) in DMEM containing 10 ml/L charcoal-stripped FBS at 37°C for 45 min. The cells were stained with crystal violet, and the cell-bound stains were quantified by determining the absorbance at 570 nm. Each bar represents the mean ± SEM (*n *= 7). Means without a common letter differ, *P *< 0.05. **(c) **Cells were serum deprived in DMEM supplemented with 10 ml/L charcoal-stripped FBS for 24 hours. Serum-deprived cells were plated onto the filter in 6.5-mm transwell inserts in 24-well plates at 5 × 10^4 ^cells/filter. Before the plating of the cells, the lower side of the transwell filter was precoated with type IV collagen. The lower chamber of the well was filled with DMEM containing 100 ml/L gelatinase-free charcoal-stripped FBS with or without various cytokines (the identical concentrations of cytokines used in Figure 6a). The cells were incubated for 16 hours. Migrated cells were stained with hematoxylin and eosin. Each bar represents the mean ± SEM (*n *= 3). Means without a common letter differ, *P *< 0.05.

We next conducted *in vitro *assays to determine the effects of the cytokines that were elevated *in vivo *by HFD on the viability, adhesion, and migration of 4T1 cells. The concentrations of cytokines used in these assays were the mean concentrations detected in the sera of the HFD-fed mice (Table 2). Among these cytokines, only sICAM-1 enhanced the viability of 4T1 cells (Figure 6a). The capacity of the 4T1 cells to adhere to strips coated with human collagen type I was increased by C5a, sICAM-1, IL-16, M-CSF, TIMP-1, and TREM-1 (Figure 6b). Additionally, TREM-1, TIMP-1, M-CSF, and sICAM-1 significantly increased the migration of 4T1 cells (Figure 6c).

## Discussion

The principal objective of this study was to determine whether dietary fat increases mammary tumor growth and metastasis as well as mammary cancer-associated mortality in obesity-resistant BALB/c mice. BALB/c mice fed on the 60% kcal-fat diet consumed less food, such that their intake of protein, vitamins, minerals, fiber, and kilocalories was only slightly increased relative to that of the control mice. Epidemiologic evidence indicates that the consumption of fiber, vegetable, and micronutrients is associated with reduced mortality in postmenopausal women diagnosed with breast cancer [28]. Other previous findings also suggested the possible benefits of a low-fat/high-vegetable diet on disease-free survival (reviewed in [29]). The present results clearly show that increased dietary fat intake, without decreasing other nutrients (except for carbohydrates) and with little effect on energy intake and weight, increases mammary cancer growth, angiogenesis, metastasis, and mortality of BALB/c mice with implanted tumors.

Xu *et al*. [30] demonstrated that a variety of inflammatory and macrophage-specific genes are upregulated dramatically in white adipose tissues of C57BL/6J mice fed a 60% kcal diet for 16 weeks. Their control and high-fat diets were probably similar to those used in this study because they were supplied by the same manufacturer. Thus, in the current study, before the injection of 4T1 mammary cancer cells, obesity-resistant BALB/c mice were fed a 60% kcal-fat diet for 16 weeks to induce chronic low-grade inflammation. We noted that the body weights were only slightly increased in the BALB/c mice fed on the HFD. At the end of the experiment, the change in body weight due to the long-term high-fat feeding was measured at only 4% after correction for differences in tumor and spleen weights. At autopsy, we were unable to detect visible adipose tissues except in the gonadal fat pad, the weight of which was increased in the HFD-fed mice. These results contrast markedly from those reported by Xu *et al*. [30], who demonstrated that HFD markedly increased the body weights of C57BL/6J mice. This discrepancy between the results was anticipated, however, because C57BL/6 mice are obesity susceptible, and BALB/c mice are obesity resistant [17]. Macrophage accumulation was noted in the adipose tissues of C57BL/6J mice fed on an HFD [31]. In this study, we noted that F4/80+ macrophage infiltration in the gonadal fat pad and the serum levels of cytokines (C5a, sICAM-1, IL-16, M-CSF, TIMP-1, and TREM-1) were elevated in HFD-fed mice (Table 2). These results indicate that dietary fat induces a low-grade inflammation in the absence of obesity, and a small increase in adipose tissue may have contributed to the induction of inflammation.

Leptin is a cytokine-like protein secreted from adipose tissue [32], and circulating leptin levels are positively associated with body weight and/or body fat [33,34]. Leptin and leptin receptor are involved in the development of normal mammary glands and in the progression of breast cancer [35-37]. In this study, we found that the serum levels of leptin were elevated in HFD-fed mice without a discernible change in body weight, indicating that a small increase in fat mass increases serum leptin levels, and the elevated leptin may have contributed to the stimulation of mammary cancer progression in these mice.

The increased expression of CD31 and VEGF in tumor tissues (Figure 3a) indicates that tumor angiogenesis increased in the HFD-fed mice. Recent evidence indicates that in addition to the interactions of cancer cells and endothelial cells, inflammatory cells also play an important role in the formation of the blood vessels that nourish a growing tumor (reviewed in [38]). Tumor-associated immune cells, including macrophages, granulocytes, and mast cells, have been shown to stimulate tumor angiogenesis (reviewed in [39]). Additionally, a significant positive correlation between the degree of angiogenesis and the number of CD68+ cells has been demonstrated in human breast carcinoma [40]. The number of CD45+ and CD68+ cells was markedly increased in the tumor tissues of HFD-fed mice (Figure 3a), indicating increased infiltration of these immune cells into tumor tissues, which possibly increased tumor angiogenesis.

In this study, we noted that blood levels of various cytokines were increased in the HFD-fed mice (Table 2). Our *in vitro *studies demonstrated that some of these cytokines stimulated the growth, migration, and adhesion of 4T1 mammary cancer cells (Figure 6). These results indicate that the increased cytokine levels may have contributed to changes in the expression of metastasis-related proteins (uPA, ICAM-1, and VCAM-1), thereby increasing metastasis. Future studies are needed to determine which types of cells secrete these cytokines in tumors, adipose tissues, the lungs, and/or the liver.

It was previously reported that adipocytes promote breast carcinoma cell growth in collagen gels by the cancer-stromal interaction [41]. Additionally, when co-injected with SUM159PT mammary adenocarcinoma cells in athymic nude mice, fully differentiated 3T3-L1 adipocytes stimulate tumor growth and lung metastasis [42]. We observed increased numbers of lipid vacuoles in the tumor tissues of HFD-fed mice (Figure 3a). Along with the tumor cells, these adipocytes may participate in the recruitment of immune cells into the tumor. The crosstalk between tumor cells, adipocytes, and immune cells within the tumors of the HFD-fed mice (Figure 3a) may have produced a broad variety of growth factors, cytokines, and chemokines, resulting in changes in the expression of proteins involved in the stimulation of cell-cycle progression of tumor cells (Ki67, PCNA, CDK2, CDK4, cyclin A, and cyclin D) and angiogenesis (VEGF) (Figure 2a-c, 3a). Additionally, the increase in new blood vessels may have supplied nutrients and growth factors for the stimulation of cell-cycle progression in addition to the stimulation of metastasis. In this study, the direct mechanism by which HFD feeding induces the expression of metastasis-regulating proteins, cyclins, and CDKs in the tumor tissues was not fully elucidated.

In this study, we noted that the weights of spleen were increased in the HFD-fed mice (Table 1), which was accompanied by increases in the serum levels of C5a, IL-16, sICAM-1, M-CSF, TIMP-1, TREM-1, and leptin (Table 2). Splenomegaly was invariably observed in 4T1-tumor-bearing mice [43-45], and correlated strongly with increased extramedullary hematogenesis and metastasis in the spleen and circulating levels of neutrophils and leukocytes [46]. It has been reported that 4T1 tumor growth is associated with increased splenomegaly or splenomegaly-associated inflammation, and various tumor-derived cytokines, such as G-CSF, GM-CSF, and IFN-γ, may be responsible for the splenomegaly in mice injected with 4T1 cells [44]. Future studies are needed to determine whether the increases in C5a, IL-16, sICAM-1, M-CSF, TIMP-1, TREM-1, and/or leptin are responsible for the increased splenic mass in HFD-fed mice.

## Conclusions

This study clearly demonstrated that dietary fat increases mammary cancer growth, metastasis, and mammary cancer-associated mortality in obesity-resistant mice. We also demonstrated that dietary fat increases the expression of proteins (Ki67, CDKs, and cyclins) involved in the regulation of cell-cycle progression as well as increases in the expression of CD31, VEGF, CD68, and CD45 in tumor tissues, thereby indicating that increases in immune cell infiltration and angiogenesis stimulate cell-cycle progression and the metastasis of tumor cells. Additionally, increases in protease (uPA) and adhesion molecules (ICAM-1, VACM) may contribute to lung metastasis in HFD-fed mice. Furthermore, increases in the serum levels of cytokines may stimulate mammary cancer metastasis in HFD-fed mice. Our results suggest that dietary fat may increase breast cancer progression, even in individuals who maintain a healthy body weight, and that replacing dietary fat with carbohydrates may delay the progression of breast cancer, thereby reducing breast cancer-associated mortality.

## Abbreviations

C5a: complement fragment 5a; CDK: cyclin-dependent kinase; DAB: 1,3 -diaminobenzidine; DMEM: Dulbecco's Modified Eagle's Medium; ELISA: enzyme-linked immunosorbent assay; FBS: fetal bovine serum; ICAM: intercellular adhesion molecule; IL: interleukin; M-CSF: macrophage colony-stimulating factor; MMP: matrix metalloproteinase; MTT: 3-[4,5-dimethylthiazol-2-yl]-2,5-diphenyltetrazolium bromide; PAI: plasminogen activator inhibitor; PCNA: proliferating cell nuclear antigen; TIMP: tissue inhibitor of matrix metalloproteinase; TREM: triggering receptor expressed on myeloid cells; uPA: urokinase-type plasminogen activator; VCAM: vascular cell-adhesion molecule; VEGF: vascular endothelial growth factor.

## Competing interests

The authors declare that they have no competing interests.

## Authors' contributions

MRC, HP, MK, and JEH carried out the majority of animal studies, including evaluation of food consumption, tumor volumes, mortality, Western blotting, immunohistochemistry, ELISA, and metastasis. JYL, HSC, and KWL were responsible for conception of the project, oversight of experiments, and training of certain participants. EJK and JHYP were responsible for conception of the project, oversight of experiments, and training of certain participants, and they drafted the manuscript. All authors read and approved the final manuscript.

## Acknowledgements

This study was supported by the Mid-career Researcher Program (grant number 2010-0006923) and the SRC program (Center for Food & Nutritional Genomics: grant number 2010-0001886) of the National Research Foundation (NRF) of Korea, funded by the Ministry of Education, Science, and Technology.

## Supplementary Material

Additional file 1 **Supplementary Table **1. Compositions of experimental diets Click here for file
