# Supplementary material for: Dietary fat increases solid tumor growth and metastasis of 4T1 murine mammary carcinoma cells and mortality in obesity-resistant BALB/c mice
Source: Breast Cancer Res. 2011 Aug 11;13(4):R78. doi: 10.1186/bcr2927 (PMC3236342; doi:10.1186/bcr2927)
Supplement: Additional file 1 — Supplementary Table 1. Compositions of experimental diets [file bcr2927-S1.DOCX]

|  | Control diet  (10 kcal % fat) | | | | High-fat diet | | | | |
| --- | --- | --- | --- | --- | --- | --- | --- | --- | --- |
|  |  |  |  |  | (45 kcal % fat) | | (60 kcal % fat) | | |
| Casein, 80 Mesh | | g | kcal | g | | kcal | | g | kcal |
|  |  |  |  |  | |  | |  |  |
|  |  | 200 | 800 | 200 | | 800 | | 200 | 800 |
| L-Cystine | | 3 | 12 | 3 | | 12 | | 3 | 12 |
|  | |  |  |  | |  | |  |  |
| Corn starch | | 315 | 1,260 | 72.8 | | 291 | | 0 | 0 |
| Maltodextrin 10 | | 35 | 140 | 100 | | 400 | | 125 | 500 |
| Sucrose | | 350 | 1,400 | 172.8 | | 691 | | 68.8 | 275.2 |
|  | |  |  |  | |  | |  |  |
| Cellulose | | 50 | 0 | 50 | | 0 | | 50 | 0 |
|  | |  |  |  | |  | |  |  |
| Soybean oil | | 25 | 225 | 25 | | 225 | | 25 | 225 |
| Lard | | 20 | 180 | 177.5 | | 1598 | | 245 | 2,205 |
|  | |  |  |  | |  | |  |  |
| Mineral mix^1)^ | | 10 | 0 | 10 | | 0 | | 10 | 0 |
| Dicalcium phosphate | | 13 | 0 | 13 | | 0 | | 13 | 0 |
| Calcium carbonate | | 5.5 | 0 | 505 | | 0 | | 5.5 | 0 |
| Potassium citrate,  1H_2_O | | 16.5 | 0 | 16.5 | | 0 | | 16.5 | 0 |
|  | |  |  |  | |  | |  |  |
| Vitamin mix^2)^ | | 10 | 40 | 10 | | 40 | | 10 | 40 |
| Choline bitartrate | | 2 | 0 | 2 | | 0 | | 2 | 0 |
|  | |  |  |  | |  | |  |  |
| Total | | 1,055.05 | 4,057 | 773.85 | | 4,057 | | 773.85 | 4,057 |

Supplemental Table 1. Compositions of experimental diets

^1)^Mineral mix for DIO series diets (g/kg): sodium chloride 259, magnesium oxide 41.9, magnesium sulfate heptahydrate 257.6, ammonium molybdate tetrahydrate 0.3, chromium potassium sulfate 1.925, copper carbonate 1.05, ferric citrate 21, manganese carbonate hydrate 12.25, potassium iodate 0.035, sodium fluoride 0.2, sodium selenite 0.035, zinc carbonate 5.6, sucrose 399.105

^2)^Vitamin mix for AIN-76A rodent diet (g/kg): vitamin A palmitate (500,000 IU/g) 0.8, Vitamin D3 (100,000 IU/g) 1, vitamin E acetate (500 IU/g) 10, menadione sodium bisulfite (62.5% menadione) 0.08, biotin, 1.0% 2, cyanocobalamin, 0.1% 1, folic acid 0.2, nicotinic acid 3, calcium pantothenate 1.6, pyridoxine-HCl 0.7, riboflavin 0.6, thiamin HCl 0.6, sucrose 978.42
